# Self-management of vaginal pessaries for pelvic organ prolapse: multi-method process evaluation, linked to the TOPSY randomised controlled trial

**DOI:** 10.1186/s12916-025-04551-8

**Published:** 2025-12-05

**Authors:** Carol Bugge, Melanie Dembinsky, Aethele Khunda, Margaret Graham, Rohna Kearney, Kirsteen Goodman, Lynn Melone, Karen Guerrero, Lucy Dwyer, Suzanne Hagen, the TOPSY team

**Affiliations:** 1 Research Centre for Health, School of Health and Life Sciences, Glasgow Caledonian University, Glasgow, G3 OBA UK; 2 Faculty of Health Sciences and Sport, University of Stirling, Stirling, FK9 4LA UK; 3 Department of Urogynaecology, The James Cook University Hospital, South Tees Hospitals NHS Foundation Trust, Marton Road, Middlesbrough, TS4 3BW UK; 4 Patient and Public Involvement Representative, c/o Glasgow Caledonian University, Glasgow, G3 OBA UK; 5 Saint Mary’s Hospital, Manchester University NHS Foundation Trust, Manchester Academic Health Science Centre, Hathersage Road, Manchester, M13 9WL UK; 6 Institute of Human Development, Faculty of Medical & Human Sciences, University of Manchester, Oxford Road, Manchester, M13 9PL UK; 7 Department of Urogynaecology, NHS Greater Glasgow & Clyde, 52 Grange Road, Glasgow, G42 9LF UK; 8 University of Glasgow, University Avenue, Glasgow, G12 8QQ UK; 9 Division of Nursing, Midwifery and Social Work, School of Health Sciences, Faculty of Biology, Medicine and Health, University of Manchester, Oxford Road, Manchester, M13 9PL UK

**Keywords:** Pelvic organ prolapse, Vaginal pessary, Self-management, Process evaluation, Programme theory

## Abstract

**Background:**

Pelvic organ prolapse negatively affects women’s quality of life globally. Vaginal pessaries are a common first-line treatment. The evidence base to support pessary self-management and to understand how it affects women’s lives is poor. This study aimed to identify the acceptability, effectiveness, fidelity to delivery, and adherence for women treated with vaginal pessary for prolapse and the healthcare professionals who treat them and how these differed between self-management and clinic-based care.

**Methods:**

Multi-method process evaluation embedded within a randomised controlled trial in 21 UK secondary care centres. Data were collected using the following: Recordings of self-management support appointments (*n* = 21) and 2-week post-support follow-up phone calls (*n* = 34), healthcare professional completed fidelity checklists of self-management support appointments (*n* = 156) and 2-week follow-up calls (*n* = 145), interviews with purposively sampled women randomised to each trial group at baseline (*n* = 36 total) and 18 months (*n* = 23), interviews with women who declined randomisation to the trial at baseline (*n* = 20) and 18 months (*n* = 18), interviews with healthcare professionals (*n* = 36), and a free-text response question in the trial questionnaire (*n* = 77 comments at baseline, *n* = 136 6 months, *n* = 127 12 months, *n* = 98 18 months).

**Results:**

Self-management was acceptable with all intervention components perceived as important for women’s self-management ability and to how the intervention worked. Women’s adherence to self-management and clinic-based care varied. Pessary-related complications negatively influenced adherence in both groups. Emotional labour from healthcare professionals in both types of pessary management was a moderator on the pathway to effectiveness. Women’s and healthcare professionals’ positive attitudes were central to successful implementation. Self-managing women expressed self-efficacy differently than those who received clinic-based care in that they were more confident in addressing common pessary problems, and their confidence grew over time. Women in the clinic-based care group had confidence but in paternalistic pessary care. Self-management and clinic-based care were delivered differently, and thus, the trial was a true test of the effectiveness of self-management.

**Conclusions:**

This is the first study to provide a programme theory for pessary self-management. Given the acceptability of self-management, the programme theory developed could be used to support the implementation of self-management in clinical practice. Further research is needed to support widespread implementation.

**Trial registration:**

ISRCTN62510577 (date of first recruitment was 16th May 2018).

**Supplementary Information:**

The online version contains supplementary material available at 10.1186/s12916-025-04551-8.

## Background

Worldwide, 5–11% of women experience symptomatic pelvic organ prolapse [1]. Prolapse refers to the herniation of the pelvic organs into the vagina or beyond, due to loss of support for the uterus, bladder, colon, or rectum. Increasing age and parity, and a family history of prolapse, are the main risk factors. Women with prolapse endure distressing symptoms including a feeling of something coming down, or bulge, in the vagina, and urinary, bowel, and sexual symptoms which negatively affect quality of life and body image [1–4]. Costs of prolapse treatment are high; for example, an estimated 9.5% of UK women have prolapse surgery [5], with the annual costs of surgery in England, France, and Germany, respectively, being €81 M, €83 M, and €144 M [6], and there is a negative impact on the economy due to absenteeism, poor occupational quality of life, and reduced productivity [7].

Vaginal pessaries are recommended as treatment for symptomatic prolapse [8–10] because they improve women’s quality of life [11, 12]. Women who use a pessary for prolapse commonly attend outpatient clinics six-monthly for pessary removal and replacement [13]. This can be inconvenient for women and costly for services [14, 15]. Worldwide, pessary management varies, with some countries promoting pessary self-management more than others [14, 16, 17]. Self-management involves individuals collaborating with healthcare professionals and/or family to support them in managing aspects of their health and/or treatment [14, 18]. Self-management is used in health conditions such as chronic obstructive pulmonary disease [19], but evidence relating to pessary self-management is lacking [14]. No effectiveness trials were identified comparing pessary self-management to clinic-based care [14, 20].

TOPSY, a multicentre randomised controlled trial (RCT) of clinical and cost-effectiveness of pessary self-management compared to clinic-based care, with a nested process evaluation, addressed this gap [21, 22], demonstrating that pessary self-management was no better and no worse for women’s quality of life than clinic-based care, led to fewer complications, and was cost-effective [23–25].

Literature searches linking pelvic organ prolapse, pessary, and qualitative or mixed methods design were undertaken at the study start, in 2021, and last updated early 2025. One scoping review [14] (with 42 included papers, only one of which was qualitative [26]) and one small qualitative study published since the review focused on pessary self-management specifically [27]. Both reported that self-management offers some advantages for women, for example offering flexibility to live their lives as they wish, but Dwyer et al. [14] identified methodological limitations across the evidence base. These, and studies about pessary use more generally, identified that women need time to adapt to pessary use but can self-manage in flexible ways once established [26–29]. Evidence is limited about context, acceptability, adherence, and fidelity to self-management. These concepts are central to understanding how an intervention can be implemented into practice [30].

The process evaluation nested within the TOPSY trial, reported herein, aimed to explain and expand on trial findings by addressing the following question:

What are the barriers and facilitators to acceptability, effectiveness, fidelity to delivery, and adherence for women treated with vaginal pessary for prolapse and the healthcare professionals who treat them, and how do these differ between self-management and clinic-based care?

## Methods

### Study design

The study is a multi-method process evaluation [22], designed using contemporary guidance to support understanding of context, implementation, and mechanisms of impact in RCTs of complex interventions [31]. New guidance was drawn upon once available [30].

The self-management intervention [32] was built on self-efficacy theory [33], normalisation process theory [34], and self-management theory [18]. Healthcare professionals who delivered the self-management intervention received training and a manual to guide delivery. Women randomised to self-management received an information leaflet and a 30-min self-management support session from a trained healthcare professional. Thereafter, a 2-week follow-up call and local telephone number were provided.

**Fig. 1:**
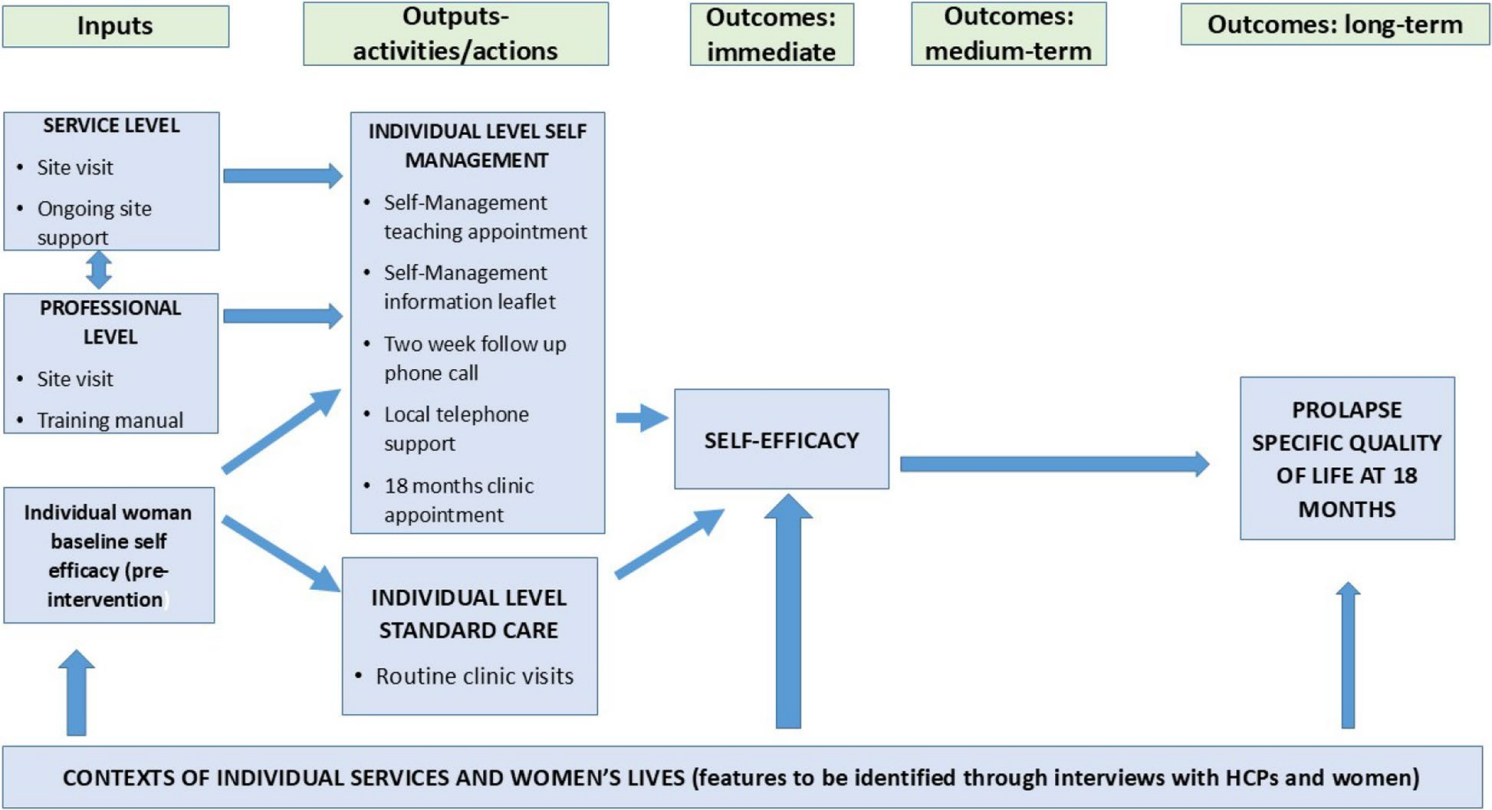
Original logic model for mechanism of action

It was hypothesised (Fig. 1) that pessary self-management would improve women’s quality of life more than clinic-based care because of the following:

- Support at service and professional levels, plus receipt of information and self-management support to women, would lead to them becoming more confident about their pessary management.
- Would improve their understanding and confidence in their self-management role
- Would enable their emotional capacity and confidence to cope with self-management, thus leading to greater improvements in condition-specific quality of life (Fig. 1).

Intervention acceptability, [pathway to] intervention effectiveness, fidelity to delivery, and adherence were investigated using the following criteria [27]:

- Contextual factors (i.e. everything beyond the intervention that may influence implementation or effect [31]): Intervention acceptability, intervention adherence, pathway to effectiveness, and self-efficacy as a potential mediating or moderating influence on that pathway. Thus, contextual factors that were personal (women), organisational (healthcare professionals and services), trial specific, and prolapse specific were explored [35].
- Implementation: Fidelity to self-management by healthcare professionals and women was considered: The extent to which the intervention was delivered (dose) and views on intervention delivery in practice (receipt and enactment).

The study aimed to develop a programme theory [30] to explain how self-management did, or did not, lead to improved prolapse-specific quality of life.

Multi-methods were used to ensure all parts of the research question were answered from varied perspectives [36], specifically as follows:

1. Audio-recordings of self-management support sessions and 2-week follow-up phone calls explored fidelity to intervention delivery from the researcher’s perspective.
2. Checklists completed by healthcare professionals at the end of self-management support sessions and after 2-week phone calls assessed healthcare professional-reported fidelity to intervention delivery.
3. Interviews with women to understand fidelity, acceptability, adherence, and contextual factors
4. Interviews with eligible women who were not randomised but were willing to be interviewed explored views on acceptability of self-management and clinic-based care, care received out with the trial, and adherence and contextual factors.
5. Interviews with healthcare professionals to understand fidelity and contextual factors
6. A trial questionnaire item (free-text response) completed by randomised women at baseline, 6, 12, and 18 months to assess experiences of acceptability, adherence, and context from the full trial population

There was patient and public involvement (PPI) throughout [37]. A Process Evaluation Management Group oversaw all aspects of the evaluation. The group was led by C. B. (academic nurse researcher who did not provide clinical care) and involved M. D. (PhD qualified, medical anthropologist), A. K. (a consultant urogynaecologist), and M. G. (a person with lived experience). The group contained three females and one male. Their backgrounds were clinically, academically, ethnically, and personally varied, and, as such, each brought that lens to the analysis. The study received ethical approval from the West of Scotland Research Ethics Service, REC 3 (17/WS/0267), on 17th February 2018 and the NHS Health Research Authority on 9th March 2018. The last interview was undertaken on 29 March 2021, and the last questionnaire was received on 31st August 2021. The trial registration is ISRCTN62510577.

### Participants and recruitment

Participants were women and people (hereafter women) recruited from 21 UK centres where pessary care was provided for prolapse [21]. Recruitment to the trial happened prior to process evaluation recruitment, with potentially eligible women identified by local clinicians who screened for eligibility, held the recruitment conversation, and randomised and consented women to the trial. Women were eligible for the trial if they were ≥ 18 years and used a pessary of any type or material, except Shelf, Gellhorn, or cube pessary (Shelf and Gellhorn pessaries are unsuitable for self-management [38], and cube pessaries *have* to be self-managed [39], making randomisation inappropriate). Two previous trials testing pessary use had high attrition rates due to pessary fitting failures, and thus, to avoid post-randomisation withdrawals, women also had to have retained the pessary for at least 2 weeks [40, 41]. Pregnant women were ineligible based upon evidence that for some women prolapse resolves following delivery [12]. Women were also excluded if they were judged by the treating healthcare professional to have limited manual dexterity, as this has been reported by women to be a barrier to self-management or a cognitive deficit that prevented self-management and/or giving informed consent [9, 42]. Finally, the self-management information was only available in English (due to budgetary constraints), and thus, women were excluded if they were unable to understand the information for the study.

Women who consented to the trial indicated on their consent form whether they would be willing to have their self-management support session and/or 2-week telephone call digitally recorded and if they were interested in taking part in an additional interview component.

The aim was to record 30 self-management support sessions and 30 follow-up calls. Purposive sampling targeted variance in centre (with at least one self-management appointment or one phone call recorded in each centre), delivering healthcare professional background (nurse/physiotherapist/doctor) and women of a range of ages. Healthcare professionals were asked to complete a checklist assessing intervention fidelity for all self-management support sessions and all 2-week phone calls.

The aim was to recruit 18 randomised women in each trial group (total *n* = 36) to be interviewed at baseline and again at 18 months post-randomisation. Purposive sampling varied on study centre, age, user status (new/existing pessary user), and treating healthcare professional background. Women who were interested in being interviewed were purposively sampled and sent an interview participant information leaflet (which included information about reasons for doing the research) with a phone call made a few days later to discuss participation in interviews. A consent form was signed prior to the first interview and verbally rechecked prior to the 18-month interview.

Women eligible for the trial who declined trial participation were invited to participate in an interview component only. They were given a participant information leaflet and an expression of interest form. The target sample was 20 women, with sampling based on convenience by the women’s decision to return the expression of interest form. Those interested were contacted by researchers for further discussion and consent.

Healthcare professionals listed on the trial delegation log were advised that they may be invited to take part in an interview. Those who had recruited trial participants and/or delivered the intervention were invited to participate in an interview, sent a participant information leaflet and consent form, and given the opportunity to ask questions. Sampling aimed for one or two participants from each centre with variance in professional background.

### Data collection

Data collection instruments for each of the six datasets are provided in Supplementary File A. Sample sizes for each component aimed to achieve information power [43]. Recruitment was complete prior to the onset of the COVID-19 lockdown.

1. *Support session/two-week phone call recordings*: With consent, a small digital recorder was placed in the consulting room or attached to the phone to record the interaction.
2. *Healthcare professional*-*completed fidelity checklists*: Checklists were developed around the core aspects of the intervention content and theory. The healthcare professional involved was asked to complete the checklist after the clinic to indicate if each item was delivered.
3. *Interviews with randomised women*: Interviews were one-on-one, semi-structured, and planned to be face to face at a place of the participants’ choosing (with M. D., who had no previous relationship with the participants). The short timeline between randomisation and self-management support session, and the national COVID-19 lockdown, meant that some interviews were online/by telephone. Interviews were undertaken at baseline (trial recruitment) and 18 months post-randomisation (end of the trial for an individual woman) to coincide with primary outcome measurement. Interview schedules were developed based on literature and with guidance from patient and public involvement team members. Interviews explored the following: Perspectives on recruitment, symptoms/ change in symptoms, experience and acceptability of clinic-based care or self-management, adherence to allocated trial group, and contextual factors that were perceived to interact with intervention effectiveness. Where a woman crossed over to receive treatment offered in the group to which she was not randomised, reasons for doing so were explored. Interviews were digitally recorded and transcribed.
4. *Interviews with non-randomised women*: Women were interviewed by telephone at baseline and 18 months later. Interviews focussed on reasons for declining trial entry, symptoms/ change in symptoms, treatment received for prolapse, and contextual factors that may interact with future service implementation.
5. *Interviews with healthcare professionals*: Semi-structured telephone interviews with recruiters in pilot centres were undertaken during the pilot study; all other interviews were towards the end of centre data collection. Recruiter interviews focussed on factors that influenced potential participant identification and recruitment, including service structures. For those involved in delivering clinic-based care and/or self-management, interviews focussed on experiences of care delivery, including variance and reasons for it, and personal/organisational contextual factors that were perceived to impact upon delivery.
6. *Free-text question in trial questionnaire*: Within the questionnaire booklet at baseline, 6, 12, and 18 months, women were asked an experiential question (“Please *write here anything you would like to tell us about your experience of pessary care”)* to explore women’s personal context across the wider sample of women in the trial.

### Data analysis

First, each of the six data sources was analysed individually to reach conclusions. Thereafter, findings were synthesised [44]. Analysis was undertaken by the Process Evaluation Management Group led by M. D. and C. B. with input from M. G. and A. K. The wider research team was blind to the findings. All recordings were transcribed verbatim and imported into NVivo12® software.

1. *Support session and two-week phone call recordings*: An a priori analytic grid was developed based on self-management theory (Supplementary File A). M. D. coded all transcripts and C. B. > 10% to assess agreement. Coding was discussed with the Process Evaluation Management Group. Descriptive statistics were used to describe the proportion of theoretical components delivered.
2. *Checklists:* Descriptive statistics were used to report the proportion of each checklist item delivered within support sessions/phone calls.
3. *, 4), 5), and 6) All interview data and free-text responses questionnaire item*: The framework approach was used [45]. First, an initial overarching thematic framework was developed based on the research questions and the theory underpinning the intervention. Second, the individual transcripts (or Excel spreadsheet download for the free-text responses) were uploaded into NVivo and read/listened to for familiarisation. Third, the framework was applied across the data and iteratively developed (M. D.). Ten percent of the data from each dataset was coded by C. B., and coding was discussed to develop the framework. M. D./C. B. discussed the data on a weekly basis. Fourth, data extracts for codes were summarised and discussed by the Process Evaluation Management Group. Fifth, the findings from each dataset were discussed. Sixth, each dataset was described and explained, focusing on context and implementation and the interactions with adherence and outcome.

To synthesise relevant data, interviews with women underwent further analysis from a case study perspective [46]. Priority was given to complete cases where there were data at both timepoints. Each case was one woman and all associated data. The case study had three tails: Randomised women who received self-management intervention, randomised women who received clinic-based care, and non-randomised women. Case summaries were written for each individual case which focussed on women’s experiences about key areas of study. All cases in one tail were gathered and consistencies/inconsistencies searched for. The tails were discussed with the Process Evaluation Management Group to identify the core findings within each tail. Tails were then compared based on the theory underpinning the intervention [46].

## Results

The trial did not find a difference in the primary outcome, prolapse-specific quality of life, between self-management, and clinic-based care [24]; therefore, data from both trial groups are presented together and distinguished only where relevant.

### Sample

In the trial, 169 women were randomised to self-management and 171 to clinic-based care [24]. The process evaluation data gathered and characteristics of the sample are presented in Table 1. The sample was varied, offering depth of women-centred and healthcare professional perspectives.

**Table 1:** Data and sample

Dataset	Data gathered (*N*)	Characteristics of the sample
1) Audio-recordings: Support sessions & 2-week follow-up calls	• *N* = 22 support sessions (data for 21 as 1 audio file was not accessible) • *N* = 34 2-week follow-up calls	At least one recording from each of the 21 trial centres
2) Checklists: Support sessions & follow-up calls (self-management group only)	• *N* = 156 support session checklists • *N* = 145 2-week follow-up call checklists	156 from a potential 169 in the self-management group^a^ 145 from a potential 156 in the self-management group^a^ Checklists received from all centres
3) Interviews: Randomised women	*Baseline* • *N* = 36 • *N* = 18 self-management • *N* = 18 clinic-based care *18 months* • *N* = 23^bc^ • *N* = 12 self-management • *N* = 11 clinic-based care	*Baseline new pessary users*^d^ • *N* = 7 self-management group • *N* = 12 clinic-based group At baseline, the interview sample showed a similar distribution in age, parity, and deprivation index to that of the main trial sample [47] *Complete cases self-management group (n* = *12)* • *N* = 5 < 65 years of age • *N* = 5 new pessary users^d^ • *N* = 1 live in most deprived area^e^ • Parity ranged from 1 to 3 births *Complete cases clinic-based care group (n* = *11)* • *N* = 4 < 65 years of age • *N* = 7 new pessary users^d^ • *N* = 3 live in most deprived area^e^ • Parity ranged from 1 to 6 births
4) Interviews: Non-randomised women	*Baseline* • *N* = 20 *18 months* • *N* = 18^bc^	*Complete cases (n* = *18)* • *N* = 5 < 65 years of age (two missing) • *N* = 2 new pessary users^d^ • *N* = 8 live in more deprived areas^e^ • Parity ranged from 1 to 4 births (four missing)
5) Interviews: Recruiters and deliverers	• *N* = 36 • *N* = 19 recruiters • *N* = 17 deliverers	At least one recruiter or one deliverer from each of the 21 centres took part 6 to > 20 years clinical experience The 19 intervention deliverers were a mix of doctors (*n* = 4), specialist nurses (*n* = 12), and physiotherapists (*n* = 1)
6) Free-text responses to question in trial questionnaire	• *N* = 77 at baseline • *N* = 136 at 6 months • *N* = 127 at 12 months • *N* = 98 at 18 months	• *N* = 35 at baseline for self-management • *N* = 42 at baseline for clinic-based care • *N* = 71 at 6 months for self-management • *N* = 65 at 6 months for clinic-based care • *N* = 64 at 12 months for self-management • *N* = 63 at 12 months for clinic-based care • *N* = 46 at 18 months for self-management • *N* = 52 at 18 months for clinic-based care

^a^Thirteen self-management support session checklists were not collected because the support session did not take place (11 women reverted to clinic-based care before the appointment, 1 woman withdrew from the study at the baseline appointment after randomisation, and 1 woman’s clinic session was cancelled due to COVID-19). Eleven follow-up phone call checklists were missing because 6 women reverted to clinic-based care shortly after the support session because they could not remove or insert their pessary, 3 women withdrew from the study, 1 woman discontinued pessary use, and 1 woman could not be reached to check she was able to remove and reinsert her pessary and so reverted to clinic-based care

^b^The data on the complete cases (data at baseline and 18 months) form the basis of this manuscript; hence, the characteristics presented mainly focus on the complete cases

^c^Thirteen randomised women were lost to follow-up, and 2 non-randomised women were lost to follow-up

^d^New pessary users were classed as those who had used a vaginal pessary for prolapse for < 3 months, and those who had used a pessary for ≥ 3 months were classed as existing users

^e^Assessment of deprivation was based on SIMD (Scottish Index of Multiple Deprivation https://simd.scot/#/simd2020/BTTTFTT/9/−4.0000/55.9000/) or IMD (Index of Multiple Deprivation https://data.cdrc.ac.uk/dataset/index-multiple-deprivation-imd)

In the randomised interview sample, two women in the self-management group crossed over to clinic-based care but were still interviewed to explore their perspectives. Due to COVID-19, five women in the clinic-based care group undertook some self-management (see the ‘Adherence’ section). These were not classed as crossovers as the women did not receive any training in how to self-manage.

### Context

Findings related to acceptability of, and adherence to, self-management and clinic-based care were described by women and healthcare professionals alongside contextual factors that influenced the pathway to effectiveness, including the hypothesised moderating factor within the mechanism of action, self-efficacy.

#### Acceptability

Regardless of whether women were self-managing, undertaking clinic-based care, or were part of the non-randomised clinical sample, they voiced that pessary self-management was acceptable.

I would, that wouldn’t be a problem em, you know, if I could self-manage it that would be fine, if not then I'd just have to, you know, get them to do it. (Vera, non-randomised, clinic-based care) .

Healthcare professionals also found it acceptable. Of particular importance for intervention acceptability was the woman–healthcare professional relationship and the availability of a telephone number to reach the local care team in case of any questions.

And knowing that if there were any problems I could ring was always useful. I think that’s quite reassuring when you know you can do that. (Liana, self-management, randomised)

Self-management also meant less travel to and from clinics and fewer costs for women.

… The service has always been good. My frustration has been that it’s an effort for me to go to the clinic because it’s a, sort of, at least an hour there and an hour back for an appointment that sometimes takes five minutes, but I don’t want to not be part of the process because I don’t want to, kind of, come out of the system. (Jasmine, self-management, randomised)

Women saw financial benefits of self-management to the NHS but stressed that freeing appointment spaces for women who really needed them was more important to them than financial benefits.

It seemed to me that if the pessary clinic, the lassie [girl] that runs the pessary clinic, if she had no…she literally had no appointments from the June right round to the following April and it seemed to me that if I self-managed then I wouldnae [would not] be taking up an appointment. And if more people did they wouldnae be taking up an appointment (Dahlia, self-management, randomised)

Self-management acceptability was facilitated by women being able to use the pessary to suit their lifestyle. Removing the pessary more frequently than every 6 months for cleaning, sexual activity, or to give the vaginal tissues a rest was reported as favourable for pessary self-management and underpinned acceptability.

If I can clean it once a week or something, then I’m quite happy just to keep it in, ‘cause, I suppose, I’m…it’s, kind of, part of me now, I don’t really want to take it out for no reason, you know, ‘cause it’s helping me so I don’t really want to take it out. But, yeah it’s a bonus that I can self-manage, definitely. (Lily, self-management, randomised)

Women in the clinic-based care group found their pessary care to be acceptable, and those in the non-randomised group generally preferred clinic-based care to self-management.

When I got the letter asking for the trial, em I just thought I could'nae, I just would hate tae insert that and go, not getting it right and trying and trying again (Azelea, non-randomised, clinic-based care)

Data from those in the non-randomised group (18/20 of whom received clinic-based care) identified that there was little difference between trial-delivered clinic-based care and clinic-based care generally provided in services. Like the self-management group, the women–healthcare professional relationship was central to acceptability for clinic-based care. Women who wanted more reassurance from a healthcare professional found self-management less acceptable due to lack of regular physical examinations.

Healthcare professionals saw the benefits of self-management to women and the NHS.

I think it's been well overdue doing a study like this to see because I think overall for patients throughout, you know speaking to other areas where self-care hasn’t been a big thing, and I think for women all over it will have a positive outcome hopefully, that more patients will be offered the self-care option. And then it takes the pressure off the health service and things a little bit if more patients are doing self-care. (54 Self-management deliverer)

#### Adherence

Women’s adherence to their allocated group was variable. One extraordinary factor that affected adherence was the COVID-19 pandemic, which led to all clinic appointments being cancelled for pessary changes for several months. Of the 11 women in the clinic-based care interview group who completed an end-of-study interview, 6 had care interrupted by COVID-19 precautions. For some clinic-based care group women (*n* = 5), this led to them undertaking some pessary self-management behaviours, but for the remainder, it did not. Those who did self-manage noted that they started to become worried that the pessary remained in situ for too long.

When we went into lockdown I did…I couldn’t go, you know, because obviously the appointments were cancelled. And so it was more like a year by the time I had my six month appointment but, you know, that's understandable. And I did change it myself…well I didn’t change it but I took it out and washed it a couple of times myself. (Veronica, clinic-based care, randomised)

Women in the self-management group experienced fewer complications than those in the clinic-based care group [24]. Experiencing complications affected adherence to trial group allocation and could extend to pessary discontinuation. Another reason for non-adherence was that some women could not gain the skills to successfully remove or insert their pessary themselves.

I’ll be able to go to the hospital, pull it out and then the lady’ll say, oh you’re fine, go home and you can wash it yourself and I’ll manage myself. But when I got there, it was a lot harder than I thought. It wasn’t like the cap, like, flexible. It’s more tough. And I couldn’t pull it. (Ivy, self-management, randomised, reverted to clinic-based care)

There were three factors that affected trial group adherence in both groups: good general health, a supportive network, and a preference for a specific treatment pathway/trial group. Good general health allowed women to attend clinic-based care appointments or to self-manage. Having good general health facilitated physical activity in both trial groups.

I do about an hour and a half of dog walking every day, and then I go on the cross trainer for half an hour three or four times a week, and I do Pilates once a week. (Daphne, clinic-based care, randomised)

Women in both trial groups valued being able to talk to family members about prolapse and felt emotionally supported in their journey. This emotional support allowed women to feel comfortable with the pessary and care pathway they were randomised to for the trial. For women assigned to the clinic-based care trial group, support was also shown by family members taking them to their appointments.

Where women preferred self-management but were randomised to clinic-based care, this acted as a barrier to group adherence. Divergence from clinic-based care adherence due to a preference for self-management varied. Some women continued to attend clinic appointments but also removed and reinserted their pessary on their own between these appointments. In contrast, others did not attend clinic appointments and fully self-managed the pessary.

[...] every day basically I change it. (Clementine, clinic-based care, randomised)

During interviews, healthcare professionals indicated that they did not veer from the teaching manual. However, recordings of self-management support sessions indicated that sometimes there was no discussion about the effect of pregnancy on pessary continuation. This is mainly due to the woman attending the appointment being post-menopausal, which the healthcare professional would know from medical records, their knowledge of the woman, and from visual cues.

#### Contextual factors that moderate the pathway to effectiveness

Teaching the necessary self-management skills to enable women’s self-efficacy required emotional labour from healthcare professionals who provided encouragement and reassurance.

Yeah, and a little bit of, sort of you know, encouragement, you know, ‘cause a lot of the ladies…some of them feel that they’re not going to be able to do it, or that they might find it difficult and then, obviously, if they do manage to do it then we’re all very pleased. (Self-Management Deliverer 14)

When talking about their experiences teaching the self-management intervention, healthcare professionals frequently commented on how they perceived participants’ responses to being instructed in pessary self-management. According to intervention deliverers, participants’ initial reactions ranged from fear and embarrassment to enthusiasm beyond their own expectations.

There’s a lot of fear initially because it’s an area that people don’t talk about a lot, it’s quite embarrassing for the patients, and a lot of them think, oh, I don’t think I could do that, that’s not something I could do. But when they actually see the pessary and how simple it is, they think oh my goodness, why did I not do that sooner, they’re really, really positive about it (Self-Management Deliverer 17)

Self-managing women expressed self-efficacy differently to those in the clinic-based group in that they felt more confident in addressing common problems with their pessary, such as discharge or slippage, on their own without the need for additional clinic appointments.

The advantage is, that in the past, sometimes it [pessary] would kind of start to slip out, especially when you’re holding the grandchildren and picking up any weights, it seems to drop down a bit. Now I can take it out and replace it properly, rather than just sort of pushing it back, if I feel like I need to (Daisy, self-management, randomised)

They also talked about their confidence changing and growing over time.

It’s a lot easier now to take it in and out to clean it. (Lily, self-management, randomised)

It just...it’s just now become, sort of, part and parcel of my daily life. (Jasmine, self-management, randomised)

The trial [24] did not find group differences in general self-efficacy, measured using the general self-efficacy scale [48], but did identify that self-managing women were more confident in their ability to insert and remove their pessary and to deal with pessary problems. Thus, there was both quantitative and qualitative evidence of differences between the groups in self-efficacy, but these did not extend to statistical differences in general self-efficacy scores.

Women in the clinic-based care group and non-randomised women wanted to be ‘looked after’ by the healthcare professional. Factors that influenced this were as follows: A long time taken to achieve a pessary with a good fit, history of recurrent urinary tract infection, a lack of knowledge about their own anatomy linked to concerns about the pessary getting lost within their body or inserted incorrectly, and women not being accepting of their body/prolapse. All of this led to comfort with a more paternalistic model of care.

I’m in denial about it slightly, so that’s why I don’t want to take the pessary out or put it back in again because I feel like I don’t like interacting with that part of my body because I feel as though it’s not, it’s let me down maybe. (Sage, clinic-based care, non-randomised)

### Implementation

Successful intervention delivery was dependent on a multitude of interrelated factors. The strongest facilitator to successful intervention delivery was staff and women’s attitudes toward pessary self-management. Healthcare professionals delivering the intervention voiced their support for pessary self-management.

It would be good for it to be rolled out nationwide, giving the confidence to the patients to look after themselves, and be in control of it. […] People that you would think maybe, oh they’re too old to do something, but age isn’t a barrier, it seems with…just if they’ve got arthritis, or can’t squeeze the pessary, that seems to be their main problem. (Self-Management Deliverer 10)

In addition, healthcare professionals described how women responded to being instructed in pessary self-management.

I think, a personal acceptance in preparation for it is more important than actually how you do it, because I don’t think how you do it, is very difficult. As just, whether you’re mentally, as a patient, prepared to go down that route. (Self-Management Deliverer 31)

Overall, healthcare professionals reported that the intervention did fit within clinical practice and local service delivery.

Those who received the self-management intervention commented that the intervention elements provided enough information, practical guidance, and follow-up for them to confidently self-manage their pessary over the duration of the trial. Clarity and content of delivery were facilitative, with all women who attended the support appointment commenting that they found the material delivered acceptable and comprehensive.

She [the nurse] showed me what to do, and I did it while I was there. It worked. And I had no problems with the changing of them. (Daisy, self-management, randomised)

Obstacles toward successful delivery of the intervention were due to women’s physical limitations such as not being able to pull the pessary out.

We changed the furniture, we got ourselves locked in and we were getting on. She just couldn’t do it […] Her hands were just so bad she could not…just any strength to pull, to do moves…even with pressure, you know…coughing and, sort of, helping…the wee soul. She persevered for so long. It’s just… But it just wasn’t to be, unfortunately. (Self-Management Deliverer 33)

There were some elements of the intervention that required refinement. The intervention prescribed that women tried to remove/reinsert their pessary during the support session, but this did not always translate to successful self-management at home. At other times, HCPs delivering the intervention opted to let women try on their own at home when the HCP felt that the woman was too anxious to try in clinic.

A lot of the patients don’t want to try and do it within the clinic, they feel rather embarrassed, and they do it at home. (Self-Management Deliverer 13)

In developing the intervention, considerable emphasis was placed on how to insert the pessary, and although pessary removal was considered, there was less emphasis on this aspect of self-management. However, self-managing women reported that they found pessary removal often more difficult than pessary insertion. Reasons given were that the shape of the pessary could not be manipulated to remove it (as it can to insert it), and hooking a fingertip around the pessary edge was not always easy to achieve.

I tried and tried with the help of the prolapse nurse and I could not remove it myself. She obviously told me exactly what to do and what position to be in and I could not do it. The thought of it was, I’m thinking I just don’t know whether I can do it, but with her guidance I could. But I’ve got very small hands and I could not hook my finger over the rim at all to remove it. And the nurse said, you’ve tried and tried, but I really couldn’t do it. So, that’s why I had to revert not to do it myself. (Heather, self-management, randomised, reverted to clinic-based care)

#### Fidelity to delivery

Fidelity to the self-management intervention delivery was demonstrated by the following: healthcare professionals reporting that, for most women, all aspects of the intervention in the support session and the 2-week phone call were delivered (Tables 2 and 3). In the support session, the only items that were reportedly delivered in under 90% of cases were checking that a woman was able to insert and remove her pessary. This may be explained by the healthcare professionals reporting that if a woman was uncomfortable practicing these skills in clinic, agreement between the woman and the healthcare professional was reached for the woman to do this at home. For the 2-week phone call, items that were delivered in under 90% of cases were asking if the women needed an additional call or an additional support session, but these items were not relevant to some women who were finding self-management easy to do. In the researcher-assessed audio-recordings, fidelity to intervention delivery was generally found to be lower than reported by the healthcare professionals in the checklist. However, the fidelity was still high except in relation to the following: the healthcare professional explaining their role, self-managing women taking responsibility for their own health, and discussing emotional management, pregnancy, and pessary storage; everything else was heard in over 75% of cases. It is possible that some of these items, such as pregnancy, were not relevant (e.g. the woman was post-menopausal); were discussed at another time-point, e.g. before the recorder was on (such as the healthcare professional explaining their role); or when the recorder was switched off (some healthcare professionals switched the recorder off when the women were inserting/removing the pessary). The analysis of the 2-week phone call audio-recordings identified lower levels of fidelity; however, it is possible that not all items were relevant to all women (e.g. women may have been asked more generally about complications and not each specific complication). Over 75% of women were asked about the items that were core to the phone call, specifically being able to remove, replace, and position the pessary.

**Table 2:** Fidelity to self-management intervention delivery in 30-min support session (checklists and audio-recordings)

	**Support session**	
*Checklist item*	***Healthcare professional completed checklist (n* = *156)***	***Researcher assessed audio-recording (n* = *21)***
Beginning		
Healthcare professional explained their role in study	99.4% (*n* = 155)	38.1% (*n* = 8)
Prolapse information provided	100% (*n* = 156)	76.2% (*n* = 16)
Information about pessary self-management. Information given about the following:		
Taking responsibility for own health	100% (*n* = 156)	38.1% (*n* = 8)
Benefits of self-management	100% (*n* = 156)	76.2% (*n* = 16)
Managing emotions	98.1% (*n* = 153)	52.4% (*n* = 11)
Teaching pessary self-management		
Discussion of pelvic anatomy	99.4% (*n* = 155)	85.7% (*n* = 18)
Discussion about type of pessary used	99.4% (*n* = 155)	90.5% (*n* = 19)
Pessary given to handle	99.4% (*n* = 155)	81.0% (*n* = 17)
Demonstrated lubrication	99.4% (*n* = 155)	90.5% (*n* = 19)
Demonstrated insertion	99.4% (*n* = 155)	85.7% (*n* = 18)
Demonstrated position	98.1% (*n* = 153)	85.7% (*n* = 18)
Demonstrated removal	99.4% (*n* = 155)	85.7% (*n* = 18)
Demonstrated cleaning	99.4% (*n* = 155)	90.5% (*n* = 19)
Discussed pessary storage	97.4% (*n* = 152)	57.1% (*n* = 12)
Instructions on how to receive replacement pessary	96.8% (*n* = 151)	76.2% (*n* = 16)
What to do if becomes pregnant	100% (*n* = 156)	19.0% (*n* = 4)
Talked about what to do if there are problems	99.4% (*n* = 155)	95.2% (*n* = 20)
Given information about additional resources	99.4% (*n* = 155)	95.2% (*n* = 20)
Talking about common pessary issues		
Talked about common issues	98.1% (*n* = 153)	81.0% (*n* = 17)
Practised pessary self-management		
Practiced self-management	Practised pessary removal — 95.5% (*n* = 149) Able to remove pessary — 85.9% (*n* = 137) Practised pessary insertion — 91.0% (*n* = 142) Able to insert pessary — 89.7% (*n* = 140)	100% (*n* = 21)
Action planning		
Reminder to remove pessary at least once over next 2 weeks	96.8% (*n* = 151)	85.7% (*n* = 18/21)
Ending		
Reminder about 2-week phone call	98.1% (*n* = 153)	90.5% (*n* = 19/21)

**Table 3:** Fidelity to self-management intervention delivery in 2-week follow-up phone call (checklists and audio-recordings)

	**Two-week phone call**	
*Checklist item*	***Healthcare professional completed checklist (n* = *145)***	***Researcher assessed audio-recording (n* = *34)***
Pessary management		
Able to remove pessary	100% (*n* = 145)	88.2% (*n* = 30)
Able to reinsert pessary	100% (*n* = 145)	76.5% (*n* = 26)
Able to position pessary	100% (*n* = 145)	76.5% (*n* = 26)
Additional call arranged	89% (*n* = 129)	20.6% (*n* = 7)
Additional support session arranged	89% (*n* = 129)	2.9% (*n* = 1)
Complications		
Vaginal discharge	98.6% (*n* = 143)	85.3% (*n* = 29)
Vaginal smell	98.6% (*n* = 143)	67.6% (*n* = 23)
Vaginal pain	98.6% (*n* = 143)	76.5% (*n* = 26)
Other pain	98.6% (*n* = 143)	67.6% (*n* = 23)
Urine infection (requiring antibiotics)	98.6% (*n* = 143)	82.4% (*n* = 28)
Urinary incontinence	98.6% (*n* = 143)	79.4% (*n* = 27)
Difficulties emptying bladder	98.6% (*n* = 143)	79.4% (*n* = 27)
Bowel incontinence	98.6% (*n* = 143)	76.5% (*n* = 26)
Difficulties emptying bowel	98.6% (*n* = 143)	73.5% (*n* = 25)
Difficulty having sex	98.6% (*n* = 143)	50.0% (*n* = 17)
Experiencing pain during sex	98.6% (*n* = 143)	44.1% (*n* = 15)
Pessary fell out	96.6% (*n* = 140)	67.6% (*n* = 23)
Non-menstrual bleeding	98.6% (*n* = 143)	76.5% (*n* = 26)
Difficulties removing pessary	95.9% (*n* = 139)	64.7% (*n* = 22)

### Programme theory of pessary self-management

**Fig. 2:**
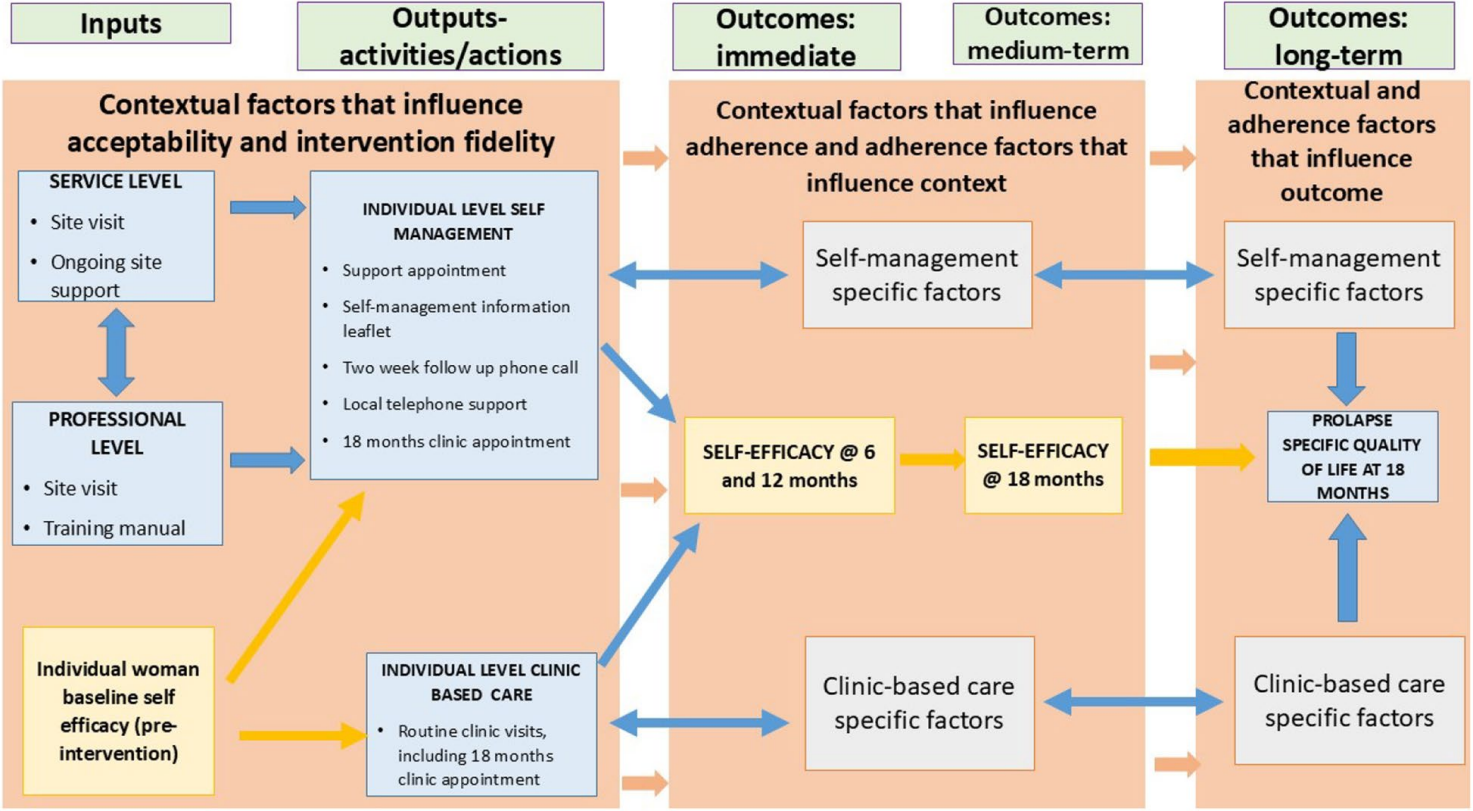
Logic model of the programme theory

Based on the above data, the programme theory was developed (Fig. 2) and is explained here. The theory starts with the intervention that is delivered (self-management or clinic-based care). All self-management intervention components were expressed as important to women’s ability to self-manage and thus were important on the pathway linking intervention and outcome. The intervention needed refinement through further instruction in pessary removal. The dominant contextual feature that influenced fidelity to self-management was women’s and healthcare professionals’ attitudes towards self-management. A key contextual feature of self-management acceptability was having a local clinic phone number that women could use if they experienced problems. Clinic-based care was valued by those who received it; in particular, support of the healthcare professional was central, along with a sense of being ‘looked after’. Following group allocation, women adhered to their allocated intervention to a greater or lesser extent over 18 months. Qualitative data demonstrated that the expression of self-efficacy by women was different between the groups. Self-managing women expressed that their ability to fit pessary care into their lives was an important contextual feature, along with their preference for self-management as a treatment pathway. Contextual factors and adherence were interwoven. In both groups, having good general health and a supportive network were important. For women in the clinic-based care group, adherence was influenced by the need for healthcare professional reassurance and enjoying the day out that going to clinic offered. The absence of complications was important to adherence and quality of life in both groups. The effectiveness of the pessary itself in symptom control, and confidence in the treating healthcare professional, was an important influence on quality-of-life outcomes. Self-managing women’s outcomes were influenced by their growing self-efficacy for managing their own pessary. For the clinic-based care group, the outcome was affected by healthcare professional reassurance via visual vaginal checks.

## Discussion

Since the research began in 2017, professional and national bodies have proposed that pessary self-management be offered to suitable women [8, 9]. The programme theory is a unique addition to the literature that may be helpful in developing practice-focused guidelines to implement such proposals. The theory demonstrates complexity with an array of interacting factors related to the intervention (such as acceptability), adherence, and context that act to influence the outcome [30]. Other programme theories related to women’s pelvic health also identify this level of complexity [49, 50].

Central to the theory is the intervention that is delivered and received [30]. There was fidelity to the self-management intervention delivery and uptake. Fidelity to clinic-based care was affected by COVID-19 but only for a small number of participants, and without affecting the statistical outcomes of the trial [24]. It is interesting that the responses to the checklists completed by the healthcare professionals indicated that more of the self-management items were delivered during teaching appointments than were heard by researchers based on the audio-recordings. Other studies have also reported this [51], and there are various possible explanations: (1) the audio-recording did not pick up all communication between the healthcare professional and the woman, (2) visual cues may have been used during teaching appointments, and (3) social desirability bias [52]. However, most self-management items were identified via audio-recordings. As such, we can be confident that the trial was a true test of the intervention, and that it can be implemented within the relevant population.

The TOPSY self-management intervention protocol offers a clear structure for self-management delivery in practice [32]. Enhanced information to support women in removing the pessary themselves is a necessary addition. Self-management acceptability for women was underpinned by having a phone number in case of problems, and future guidelines may wish to include this as an important element of pessary self-management services.

As part of a pragmatic trial, adherence to the intervention was monitored and not controlled [53]. Ruffolo et al. [42] did not find that self-management influenced adherence more than clinic-based care, but other studies have noted self-management as a positive factor in pessary continuation [54]. Qualitative data outlined above may explain some of this variance, with adherence varying for women in both groups for various reasons (e.g. personal and organisational contextual factors) [32], which was also seen in a study of women with urinary incontinence [49]. To implement self-management in practice, practitioners may wish to explore women’s personal context as part of a counselling conversation about the suitability of pessary self-management. The acceptability of self-management to women and to healthcare professionals, the high levels of adherence [55], and the cost-effectiveness [23] support a move towards clinical implementation.

It is clear from the programme theory that achieving quality-of-life impact is complex and influenced by several interacting contextual factors (e.g. self-efficacy, general health, and woman-healthcare professional dynamics). Based on self-management theory, self-efficacy was a hypothesised mediating factor in the mechanism of action [32]. The trial data suggested, and process evaluation data support, that general self-efficacy did not differ between self-management and clinic-based care groups, but self-managing women were more confident specifically in inserting and removing their pessary and in managing pessary problems [24]. Process evaluation findings also support that women who self-managed reported positive experiences that they linked to their confidence in being able to manage their pessary to suit their lives and lifestyles [27], and the way self-efficacy was expressed in relation to self-management was different than for clinic-based care. One other small study investigated self-efficacy and pessary self-management [56], giving self-managing women verbal instruction and an education leaflet. Women were more confident at 1 week and 3 months later, but the study had no clinic-based comparison group. Exploring the broader self-management literature revealed that the links between self-management and self-efficacy are uncertain. For example, self-management for people following stroke was reported to improve self-efficacy (low quality evidence), but digital interventions for people with chronic obstructive pulmonary disease did not (very uncertain evidence) [57, 58]. Future studies on self-management may wish to consider whether measures that explore specific features of self-efficacy relevant to the programme are available, as generic measures may not identify changes.

The self-management intervention development referenced normalisation process theory (NPT) to inform future clinical implementation [32, 34, 59]. All four components that underpin NPT were evident in the data (coherence, cognitive participation, collective action, and reflexive monitoring) [29, 49]. The understanding of these NPT concepts and how they influence practice helps support widespread clinical implementation. However, more formal implementation research is needed, exploring the NPT concepts in the context of pessary self-management while drawing upon additional theory and implementation frameworks [60, 61].

### Study limitations

The study excluded women who did not have a comprehensive understanding of the English language (the materials for the trial and intervention were only available in English), and the sample from whom data were gathered was predominately White British. This does seem to reflect the select population of women who seek treatment for prolapse in UK-based secondary care settings [62]. Although epidemiological studies identify differences in the incidence of prolapse across ethnicities, prolapse is a condition experienced by women from all ethnic groups [1]. The lack of ethnic diversity, therefore, extends beyond the study to women’s health-seeking behaviours for prolapse. Further worldwide research is needed to identify why those from underrepresented groups may be less likely to seek healthcare for prolapse and to expand/adapt the programme theory for pessary self-management to represent the needs of women from the global majority and those who are not fluent English speakers.

The study also excluded women with insufficient cognitive capacity to consent or to follow pessary self-management instruction. Guidelines currently advise that these women do not self-manage [63].

As a result of COVID-19, some women in the clinic-based care group who were interviewed did choose to temporarily self-manage their pessary. Results from the trial (Hagen et al., 2023) demonstrate that removal of the small number of women (26 of 171) who were affected did not influence the main intention-to-treat analysis findings. As such, we can be clear that COVID-19 did not influence the trial results. One strength of having the process evaluation data is that it is possible to be transparent about the reasons why a small number of women were not adherent to their clinic-based care pathway during COVID-19 and for other reasons.

Not all women who were interviewed completed the 18-month data collection. It is possible that those who were not interviewed at 18 months were different from those who were. For example, those who found self-management less acceptable or were not adherent. However, the voices of women are represented by those who crossed over from self-management to clinic-based care and women in the clinic-based care group who undertook some self-management. These women, along with those in the non-randomised interview group, still found the principle of offering self-management acceptable. Therefore, self-management does seem to be an acceptable treatment pathway for those who are able and willing to self-manage.

As a UK-based study, the contexts identified may not apply to other service structures. The UK is a Nationalised Health Service that is free to service users; therefore, financial barriers to care, for example, were not identified. It is possible that in private services healthcare professionals may not want to promote self-management in case it decreases appointments and hence income. Studies exploring self-management contexts in other countries would be valuable.

## Conclusions

Self-management of vaginal pessaries is an acceptable pathway for pessary care for women with prolapse. With minor changes to the intervention tested, self-management could be used in practice following the guidance within the programme theory which identifies the contextual factors that influence that pathway. The process evaluation was one part of a study that also contained a randomised controlled trial and a cost-effectiveness evaluation [23, 24]. In combination, these three studies give a clear picture of pessary self-management effectiveness, cost-effectiveness, and how the intervention may work to achieve an effect.

## Supplementary Information

Supplementary Material 1: Supplementary File A: A1. Support session healthcare professional completed checklist. A2. Two-week follow up call healthcare professional completed checklist. A3. Interview schedule for randomised women at baseline. A4. Interview schedule for randomised women at 18 months. A5. Interview schedule for non-randomised women at baseline. A6. Interview schedule for non-randomised women at 18 months. A7. Interview schedule for healthcare professional recruiters. A8. Interview schedule for healthcare professional intervention deliverers. A9. Support session a priori coding frame. A10 Two-week follow up call a priori coding frame.

## Data Availability

The datasets generated and/or analysed during the current study are not publicly available due privacy and ethical reasons but are available from the corresponding author on reasonable request.

## Abbreviations

NPT: Normalisation process theory
PPI: Patient and public involvement
RCT: Randomised controlled trial
TOPSY study: *T*reatment *O*f *P*rolapse with *S*elf-care pessar*Y* (a multicentre randomised controlled trial, with nested process evaluation, to test the clinical and cost-effectiveness of self-management of vaginal pessaries to treat pelvic organ prolapse, compared to standard care to improve women’s quality of life)
UK: United Kingdom
