# Supplementary material for: Self-management of vaginal pessaries for pelvic organ prolapse: multi-method process evaluation, linked to the TOPSY randomised controlled trial
Source: BMC Med. 2025 Dec 5;24:19. doi: 10.1186/s12916-025-04551-8 (PMC12797790; doi:10.1186/s12916-025-04551-8)
Supplement: Supplementary file 1 — Supplementary Material 1: Supplementary File A: A1. Support session healthcare professional completed checklist. A2. Two-week follow up call healthcare professional completed checklist. A3. Interview schedule for randomised women at baseline. A4. Interview schedule for randomised women at 18 months. A5. Interview schedule for non-randomised women at baseline. A6. Interview schedule for non-randomised women at 18 months. A7. Interview schedule for healthcare professional recruiters. A8. Interview schedule for healthcare professional intervention deliverers. A9. Support session a priori coding frame. A10 Two-week follow up call a priori coding frame. [file 12916_2025_4551_MOESM1_ESM.docx]

**Supplementary Materials**

| A1 | Support session healthcare professional completed checklist |
| --- | --- |
| A2 | Two-week follow up call healthcare professional completed checklist |
| A3 | Interview schedule for randomised women at baseline |
| A4 | Interview schedule for randomised women at 18 months |
| A5 | Interview schedule for non-randomised women at baseline |
| A6 | Interview schedule for non-randomised women at 18 months |
| A7 | Interview schedule for healthcare professional recruiters |
| A8 | Interview schedule for healthcare professional intervention deliverers |
| A9 | Support session a priori coding frame |
| A10 | Two-week follow up call a priori coding frame |

**A1**

| Participant ID | | | | | | 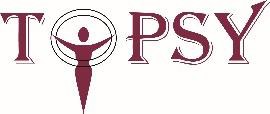 | **Self-management teaching session**  **CRF-05** |
| --- | --- | --- | --- | --- | --- | --- | --- |
|  |  |  | |  |  |  |  |
| **Note to researcher who completes the intervention training**  **This form aims to help us understand what happens in the self-management teaching sessions. We know that what you do will vary with individual women according to their needs. We are simply aiming to find out what each woman receives within her self- management teaching session and to help us understand the inevitable variability that occurs across women who are taught to self-manage.**  **Please complete the questions below in the woman who are randomised to self-management ONLY** | | | | | | | |
| Date of Self- management teaching session | | | D D Y Y Y Y | | | | |

| M | M | M |
| --- | --- | --- |

| **Beginning** | | | | | |
| --- | --- | --- | --- | --- | --- |
| Have you explained your role in the TOPSY study  Information on pelvic organ prolapse given (including discussion of prolapse as common condition) | | | | **YES**  **YES** | **NO**  **NO** |
| Information about self-management (for pessary care) | | | | | |
| Talked to woman about taking care of her own health | | | **YES** | | **NO** |
| Talked about pessary | benefits of self-management | of | **YES** | | **NO** |
| Discussed managing emotions such as fear or anxiety | | | **YES** | | **NO** |
| **Teach pesssary Self-Management** | | | | | |
| Discussed anatomy of vagina, pelvis | | | **YES** | | **NO** |
| Talked about the type of pessary the woman is using and how it works | | | **YES** | | **NO** |

| Woman provided with pessary to handle | **YES** |  |  | **NO** |  |
| --- | --- | --- | --- | --- | --- |
| Demonstrated how to apply lubrication | **YES** |  |  | **NO** |  |
| Demonstrated insertion of pessary | **YES** |  |  | **NO** |  |
| Demonstrated positioning of pessary | **YES** |  |  | **NO** |  |
| Demonstrated removal of pessary | **YES** |  |  | **NO** |  |
| Demonstrated cleaning of pessary | **YES** |  |  | **NO** |  |
| Discussed storage of pessary | **YES** |  |  | **NO** |  |
| Informed about how to receive a replacement pessary | **YES** |  |  | **NO** |  |
| Informed about discontinuation of pessary use if woman becomes pregnant | **YES** | **NO** |  |  | **N/A** |
| Discussed what to do in case of problems | **YES** |  |  | **NO** |  |
| Given information about additional resources (support phone number, link to video) | **YES** |  |  | **NO** |  |
| **Talking about common issues** | | | | | |
| Did you talk about common issues women may have with a pessary, such as discharge or what to do when menstruating (if appropriate)? | **YES** |  |  | **NO** |  |
| **Practice Pessary Self-Management** | | | | | |
| Has the woman practiced pessary removal? Has the woman practiced pessary insertion? Woman was able to remove her pessary Woman was able to insert her pessary | **YES YES**  **YES YES** |  |  |  | **NO NO**  **NO NO** |
| **Action Planning** | | | | | |
| Talked about removing, cleaning and reinserting pessary at least once over next 2 weeks | **YES** |  | **NO** |  |  |

| Discussed with woman that after this she can  remove/reinsert pessary to accommodate her lifestyle | | **YES NO** | | |
| --- | --- | --- | --- | --- |
| **Ending** | | | | |
| Check if any further questions  Centre support line phone number given Inform woman of 2 weeks follow up phone call | | **YES** |  | **NO** |
|  |  | **YES NO**  **YES NO** | | |
| If it was appropriate to omit any part of the above content please explain: | | | | |
| If it was appropriate to add something that was not part of the above content please let us know what was added: | | | | |
| Any further comments: | | | | |
| **Self-management teaching delivered by SIGN & DATE** | | | | |
|  | | | | |
|  |  | | | |
| PRINT NAME | SIGNATURE | | | |
| D D Y Y Y Y | | | | |

| M | M | M |
| --- | --- | --- |

| **Signature (Person who added Information to study database – TOPSY OFFICE USE)** | |
| --- | --- |
|  |  |
| PRINT NAME | SIGNATURE |
| D D Y Y Y Y | |

Send a COPY of this from to the address below or email to [TOPSY@gcu.ac.uk](mailto:TOPSY@gcu.ac.uk) with the pt ID as the file and email title. File the original in the Investigator Site File.

| M | M | M |
| --- | --- | --- |

TOPSY GLASGOW STUDY OFFICE NMAHP Research UnitGovan Mbeki building, Level 6, Glasgow Caledonian University, Cowcaddens Road, Glasgow G4 0BA,

**A2**

| Participant ID | | | | | 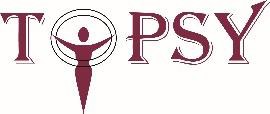 | **Self-management 2 week follow up call CRF**  **CRF-06** |
| --- | --- | --- | --- | --- | --- | --- |
|  |  |  |  |  |  |  |

| M | M | M |
| --- | --- | --- |

| **To be completed for women in the SELF-MANAGEMENT group only**   - The woman should be telephoned **2 weeks AFTER** the self-management teaching session and this CRF-06 should be completed. - If the woman has not changed the pessary at 2 weeks, ask her to do so over the next **1 week**. Call her again after one week to check if she has managed to change her pessary and complete a new CRF-06. Where a woman has experienced difficulty that requires assessment or where the woman has not changed the pessary by the time of the second phone call, offer a second self- management teaching appointment. - If, after this second appointment, the woman is unable to self-manage or does not wish to do so, give the woman the choice to transfer to standard pessary care. | |
| --- | --- |
| Date of follow-up call | D D Y Y Y Y |

| **PART A: Pessary management** | **PLEASE TICK** | |
| --- | --- | --- |
|  | **YES** | **NO** |
| **A1.** Has the woman removed, cleaned and re-inserted her pessary?  If NO, answer A2-A4. If YES, go straight to Part B. |  |  |
| **A2.** Was an additional call arranged? |  |  |
| **A3.** Was an additional training session arranged? |  |  |
| **A4.** Did the woman return to standard care? (If YES, complete a change of  status form) |  |  |
| **PART B: Complications**  **Does the woman have any complications listed below?** There may be more than one complication so for clarity please tick Yes or NO for each option. | **PLEASE TICK** | |
|  | **YES** | **NO** |
| Bothersome vaginal discharge |  |  |
| Bothersome vaginal smell |  |  |
| Vaginal Pain |  |  |
| Other Pain (please specify location and any other details below) |  |  |
| Urine infection (requiring antibiotics) |  |  |
| Urine incontinence (leakage) |  |  |
| Difficulty emptying bladder |  |  |
| Bowel incontinence (leakage) |  |  |

| Difficulty emptying bowel |  |  |
| --- | --- | --- |
| Difficulty having sex |  |  |
| Pain during sex |  |  |
| Pessary fell out |  |  |
| Vaginal non-menstrual bleeding |  |  |
| Unable to remove pessary |  |  |
| Difficulty removing pessary |  |  |
| Advice on pessary removal and re-insertion |  |  |

| M | M | M |
| --- | --- | --- |

| **PART C: FINAL OUTCOME OF CALL** | **PLEASE TICK** | |
| --- | --- | --- |
|  | **YES** | **NO** |
| **C1.** Woman is ok to self-manage without further support (This should only be ticked YES if A2-A4 were ALL marked as NO. |  |  |

**Note to researcher**

- Check whether the woman has any questions.
- Ensure the woman has the appropriate clinic telephone number to call in case of any problems.
- Remind the woman to remove, clean and reinsert the pessary at least every 6 months

| **Signature (person who completed the telephone call)** | |
| --- | --- |
|  |  |
| PRINT NAME | SIGNATURE OF SCREENER |
| D D Y Y Y Y | |

| **Signature (Information added to study database)** | |
| --- | --- |
|  |  |
| PRINT NAME | SIGNATURE OF SCREENER |
| D D Y Y Y Y | |

**Send a COPY of this from to the TOPSY Glasgow office or email to** [**TOPSY@gcu.ac.uk**](mailto:TOPSY@gcu.ac.uk) **with the pt ID as the file and email title. File the original in the Investigator Site File.**

| M | M | M |
| --- | --- | --- |

**A3**


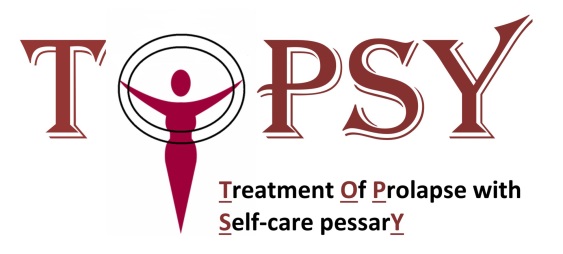


**An interview study with women who have pelvic organ prolapse about their views on treatment**

**Baseline Interview (Interview A)**

## **Introduction to study and self**

Thank you for agreeing to do the interview. We appreciate your willingness to help with the TOPSY study. The interview is about women’s experience of prolapse and prolapse-related care. I am *name,* one of the researchers on the TOPSY study.

## **Consent**

Go over study and what is involved. Do you have any questions for me? Are you still happy to be interviewed and for the interview to be recorded? If yes to all – ask to sign consent.

## **Introduction to interview**

Today’s interview is about your experience of prolapse and what you hope for the treatment you are about to have. It will take approximately 30 minutes.

## **Ice breaker**

How is your health generally?

## **Perspective on recruitment**

- How did you find completing the baseline questionnaire?

## **Woman’s experience of prolapse and symptoms**

- When did you start experiencing prolapse?
- What do you think caused it?
- What symptoms do you have? (prompt for pain, feeling something coming down, urinary, bowel, sexual symptoms)
- To what extend do symptoms bother you? Where and when most/least bothersome and why (context/situation specific)
- Progression over time (both what has happened and what they think will happen)
- External influences
- Where get information about prolapse? (what you seek/ what you get given / sources e.g. web, magazine, other women etc.)
- Do you have any children?
- Who else knows about your prolapse? (family, friends, ask about children)
- Explore support from others, who/ what support offered?
- Does your prolapse affect others close to you (family, friends)?
- What made you seek help?

## **Current Self-Care**

- Do you do anything to manage your prolapse?
- Does anything you do make it worse?
- Anything tried in the past
- Routines (such as going to the toilet before leaving house/ knowing where toilets are)
- Medication
- Exercise
- Pessary
- Confidence in managing prolapse (self-efficacy)

## **Expectations of Treatment**

- What do you understand / know about treatment for prolapse? (prompts: PFMT, pessary, surgery, anything else)
- What do you expect the recommended treatment to be? (probe pessary use, self-management/standard care)
- Can you describe what you think the treatment will be like for you? (practical, clinical, feelings)
- What do you hope to get from treatment? (try to identify main outcome wants to change/ why this or these outcome(s) most important to her)
- Expect to happen  (processes of health care and do they think/expect improvement or not)
- Want to happen (in this treatment and do they want an operation instead or afterwards)
- Anticipate anything that might influence treatment (e.g. ability to attend/change pessary, remembering to attend appointments/self-care, external influences such as work/home, service)
- What will make it easier for you / what will make it more difficult for you?

## **Closure**

Thank you for talking with me today.  The interview we have recorded will be removed from the recording device as soon as possible and stored securely.  When the interview is typed up (transcribed), all identifying information will be removed.  We will study the information you have given us alongside that given by other women.  We would like to speak with you again in 18 months’ time when we will talk about how you have got on with the treatment.  We can come to your home or to the clinic, whichever you would prefer.  I will call you to make that appointment; confirm consent to call.

**A4**


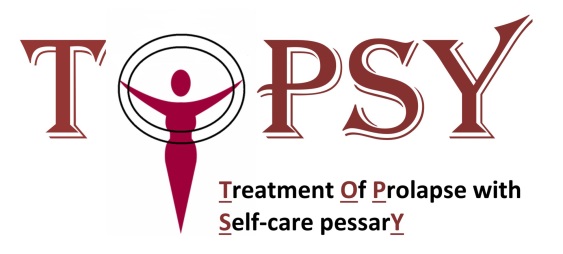


**An interview study with women who have pelvic organ prolapse about their views on treatment**

**Follow-up Interview (Interview C)**

## Re-introduction to study and self

Thank you for agreeing to see me again. We greatly appreciate the help you are giving with the TOPSY study. To recap the TOPSY interview study is about women’s experience of prolapse and how they have got on with the treatment they have been given. This interview will focus on the last 18 months (this is the last time we spoke). I am xxx, one of the researchers on the TOPSY study.

## Consent

Today’s interview will take approximately one hour. Do you have any questions for me before we start? Are you still happy to be interviewed and for the interview to be recorded? If yes to all – ask to sign consent.

## Ice breaker

How have you been generally since we last spoke?

## Symptoms [maintain focus on comparison to 18 months ago]

- Extent of prolapse symptoms now
- Comparison of symptoms now to 18 months ago (i.e. pre-treatment)
- Perceptions of stages of change (i.e. when noticed, what changed)
- Why do you think things have changed/not changed? Probe for things in relation to:
  - social (e.g. family support)
  - intervention (e.g. were there things about the intervention that the person thinks are related to change)
  - confidence to change pessary (self-efficacy)/attend appointments
  - lifestyle/ self-management (e.g. fluid, dietary changes)

## Intervention

- General views on intervention (How did you find the treatment you received?)
- *Probe about self-management of pessary – features positive, features not so helpful*
  - *What was it like being taught how to change your pessary*
  - *How did you get on doing it yourself? (check confidence in technique) (mastery)*
- What was most helpful about the treatment? (probe: self-management/ therapist or nurse etc.)
- Did you have any concerns about treatment (probes: self-management/ therapist or nurse etc.)
- Anything change about treatment? Why?
- Experience of service delivery context (e.g. appointment system, privacy ….)
- Explore perceptions of relationship with therapist
- Anything outside the service delivery that influenced experience of treatment – external influences

## Appointments

- Adherence to appointments (did you manage to attend all the appointments)
  - Opinions on number of appointments (too many/ too few)
- Factors that affected ability to attend/ not attend scheduled appointments
  - Social influences (e.g. family commitments)
  - Environmental influences (e.g. ability to travel)
  - Your own confidence (self-efficacy) influences?

## Self Care

- Self-management undertaken at home:
  - *Experience of changing pessary at home ( detail – where, when, how often)*
  - What was easy? What was difficult?
- Any other ways you manage prolapse?
- *Factors influencing adherence to self-management:*
  - *Things that helped you stick with self-management*
  - *Things that stopped/hampered self-management*
  - *Did you manage to form a routine for changing your pessary? What was it? How did it work for you? [questions about maintenance]*
  - *Was there anyone to help you stick to your routine? Or did anyone hinder your ability to self-manage?*
  - *Other social influences (such as work commitments etc)*
  - *Other environmental influences (such as privacy at home etc)*
  - *How is your confidence to change your pessary now? Has it changed over time?*
  - *Do you plan to continue with the self-management? Explore what will do? How will do?*

## Links between intervention and outcome (if not been explicitly covered through previous content)

- Links between intervention and outcome: what are they perceived to be; how do they make a difference; why do they make a difference?
- What was it like to take part in the research study (more generally)

## Completion of study questionnaires

How did you find completing the study questionnaires?

Did you use the provided Aide memoire to help you complete the follow-up questionnaires?

How useful did you find it?

## Closure

Thank you for talking with me today. The interview we have recorded will be removed from the recording device as soon as possible and stored securely. When the interview is typed up, all identifying information will be removed. We will study the information you have given us alongside that given by other women. Thank you again for being involved in the TOPSY study.

**A5**


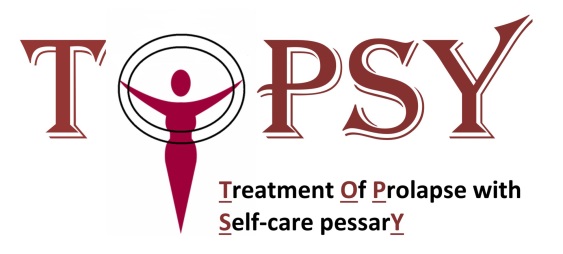


**An interview study with women who have pelvic organ prolapse about their views on treatment**

**Baseline Interview (Interview B)**

## **Introduction to study and self**

Thank you for agreeing to do the interview. We appreciate your willingness to help with the TOPSY study. The interview is about women’s experience of prolapse and prolapse-related care. I am *name,* one of the researchers on the TOPSY study.

## **Consent**

Go over study and what is involved. Do you have any questions for me? Thank you for sending us the consent form. Are you still happy to be interviewed and for the interview to be recorded? If yes to all – get verbal consent and begin interview.

## **Introduction to interview**

Today’s interview is about your experience of prolapse and what you hope for any future treatments you may have. It will take approximately 30 minutes.

## **Ice breaker**

How is your health generally?

How old are you?

Do you have any children?

## **Woman’s experience of prolapse and symptoms**

- When did you start experiencing prolapse?
- What do you think caused it?
- What symptoms do you have? (prompt for pain, feeling something coming down, urinary, bowel, sexual symptoms)
- To what extend do symptoms bother you? Where and when most/least bothersome and why (context/situation specific)
- Progression over time (both what has happened and what they think will happen)
- External influences
- Where get information about prolapse? (what you seek/ what you get given / sources e.g. web, magazine, other women etc.)
- Who else knows about your prolapse?
- Explore support from others, who/ what support offered?
- Does your prolapse affect others close to you (family, friends)?
- What made you seek help?

## **Current Self-Care**

- Do you do anything to manage your prolapse?
- Does anything you do make it worse?
- Anything tried in the past
- Routines (such as going to the toilet before leaving house/ knowing where toilets are)
- Medication
- Exercise
- Pessary
- Confidence in managing prolapse (self-efficacy)

## **Expectations of Treatment**

- What do you understand / know about treatment for prolapse? (prompts: PFMT, pessary, surgery, anything else)
- What do you expect the recommended treatment to be? (probe pessary use, self-management/standard care)
- Can you describe what you think the treatment will be like for you? (practical, clinical, feelings)
- What do you hope to get from treatment? (try to identify main outcome wants to change/ why this or these outcome(s) most important to her)
- Expect to happen  (processes of health care and do they think/expect improvement or not)
- Want to happen (in this treatment and do they want an operation instead or afterwards)
- Anticipate anything that might influence treatment (e.g. ability to attend/change pessary, remembering to attend appointments/self-care, external influences such as work/home, service)
- What will make it easier for you / what will make it more difficult for you?

## **Reasons for declining participation**

- Would you mind telling me how you felt about being asked to take part in the TOPSY main trial?
- What influenced your decision to not participate in the trial?
- **How did you feel about the recruitment process for the interview study?**

## **Closure**

Thank you for talking with me today.  The interview we have recorded will be removed from the recording device as soon as possible and stored securely.  When the interview is typed up (transcribed), all identifying information will be removed.  We will study the information you have given us alongside that given by other women.  We would like to speak with you again in 18 months’ time when we will talk about how you have got on with the treatment.  We can come to your home or to the clinic, whichever you would prefer.  I will call you to make that appointment; confirm consent to call.

**A6**


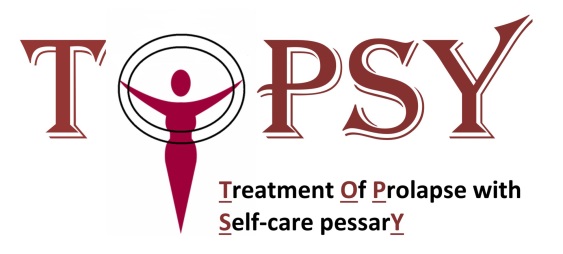


**An interview study with women who have pelvic organ prolapse about their views on treatment**

**18 month Follow-up Interview**

## Re-introduction to study and self

Is this an OK time to call? Is there another time I can call back?

Thank you for agreeing to see me again. We greatly appreciate the help you are giving with the TOPSY study. To recap the TOPSY interview study is about women’s experience of prolapse and how they have got on with the treatment they have been given. This interview will focus on the last 18 months (this is the last time we spoke). I am [name] one of the researchers on the TOPSY study.

## Consent

Today’s interview will take approximately one hour. Do you have any questions for me before we start? Are you still happy to be interviewed and for the interview to be recorded? If yes to all – get verbal consent and begin interview.

## Ice breaker

How have you been generally since we last spoke?

## Symptoms [maintain focus on comparison to 18 months ago]

- Extent of prolapse symptoms now
- Comparison of symptoms now to 18 months ago (i.e. pre treatment)
- Perceptions of stages of change (i.e. when noticed, what changed)
- Why do you think things have changed/not changed? Probe for things in relation to:
  - social (e.g. family support)
  - any treatment they have received
  - lifestyle/ self-management (e.g. fluid, dietary changes)

## Treatment received for prolapse

- What treatment have you received for you prolapse?
- General views on treatment (probes: positive features, not so helpful features)
- Anything change about treatment? Why?
- Experience of service delivery context (e.g. appointment system, number of appointments, privacy)
- Explore perceptions of relationship with healthcare professionals
- Anything outside the service delivery that influenced experience of treatment – external influences:
- Social influences (e.g. family commitments)
  - Environmental influences (e.g. ability to travel to appointments)
  - Your own confidence (self-efficacy) influences?

## Current Self-Care

- Do you do anything different to manage your prolapse (compared to 18 months ago)?
- Anything tried in the past
- Routines (e.g. going to the toilet before leaving house/ knowing where toilets are)
- Medication
- Exercise
- Pessary
- Confidence in managing prolapse and pessary(self-efficacy)

## Links between intervention and outcome (if not been explicitly covered through previous content)

- Links between treatment and outcome: what are they perceived to be; how do they make a difference; why do they make a difference?

## Closure

Thank you for talking with me today. The interview we have recorded will be removed from the recording device as soon as possible and stored securely. When the interview is typed up, all identifying information will be removed. We will study the information you have given us alongside that given by other women. Thank you again for being involved in the TOPSY study.

**A7**


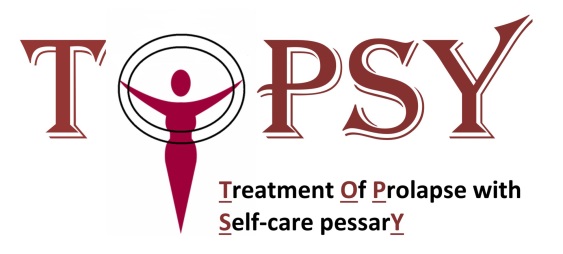


**An interview study with healthcare professionals who have assisted with recruitment for the TOPSY study**

## Introduction

Thank you for agreeing to talk with me. We greatly appreciate your willingness to help with this part of the TOPSY study, which is about how you found recruiting of women for the TOPSY trial. I am xxx, one of the researchers on the TOPSY study.

## Consent

Go over study and what is involved. Do you have any questions for me? Are you still happy to be interviewed and for that interview to be audio-recorded? If yes to all – get verbal consent and begin interview.

## Introduction to interview

Today’s interview is about your experience of being a health professional recruiting women for TOPSY. It will take approximately 30 minutes.

## Ice breaker

How did you first get involved in this area?/ When did you start working in this area?

## Questions about your role as a health professional

- Number of years of professional experience (including any overseas)
- Number of years of treating patients with prolapse
- Speciality of role (in this treatment area and in terms of profession e.g. physio, nurse advisor and scope of practice). Proportion of work time spent on patients with prolapse.

## Health professionals perceptions about recruiting women for the TOPSY study

- Please will you tell me about what went well with recruitment
- Please will you tell me about what didn’t go so well
- What do you think influences recruitment? (probes: service structure; who to approach)
- Was there ever a time where manual dexterity was raised as a potential obstacle during a recruitment discussion? (Follow-on: How did you address this?)
- How did you approach recruitment for the non-randomised interview study? (white envelopes)

## Following the study protocol

- Please will you tell me what it was like using the study intervention protocols
  - How easy/ difficult were they to follow
    - Did you have to make any variations? What were they?
    - What was it like to complete the paperwork?
    - What was it like to have you session audio-recorded (if it was)
- Please tell me about the training you received prior to taking part in TOPSY?
  - What went well?
  - What went less well?
  - What could have been improved?
- Please will you tell me about the support you received during the study
  - Was this sufficient?
  - What could have been done differently?
- What might you continue doing with women even though the trial is over?

## Expectations of about being in the study

- Please tell me about whether being involved with this study met your expectations?
  - Would you take part in future research studies as a health professional recruiting participants (with this type of protocol)?
  - Would you recommend involvement in research to other health professionals?

## Closure

Thank you for talking with me today. The interview we have recorded will be removed from the recording device as soon as possible and stored securely. When the interview is typed up, all identifying information will be removed. We will study the information you have given us alongside that given by other health professionals. Thank you again for being involved in the study.

**A8**


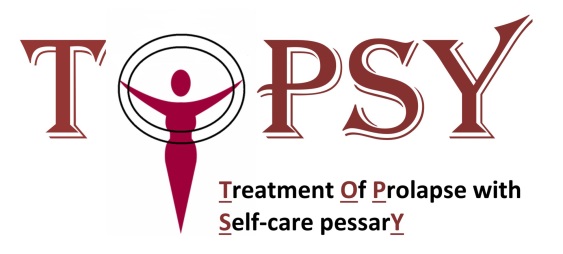


**An interview study with healthcare professionals who delivered the intervention/standard care in the TOPSY study**

## Introduction

Thank you for agreeing to talk with me. We greatly appreciate your willingness to help with this part of the TOPSY study, which is about how you found delivering the TOPSY interventions and following the trial intervention protocol. I am [researcher name], one of the researchers on the TOPSY study.

## Consent

Go over study and what is involved. Do you have any questions for me? Are you still happy to be interviewed and for that interview to be audio-recorded? If yes to all – check consent has been received, verbal confirmation of consent and begin interview.

## Introduction to interview

Today’s interview is about your experience of being a health professional delivering the treatments for TOPSY. It will take approximately 30 minutes.

## Ice breaker

- How did you first get involved in working with women who have prolapse?
- When did you start working with women who use a pessary as prolapse treatment?

## Questions about your role as a health professional

- Number of years of professional experience (including any overseas)
- Number of years of treating patients with prolapse
- Speciality of role (in this treatment area and in terms of profession e.g. physio, nurse advisor and scope of practice). Proportion of work time spent on patients with prolapse problems.
- What was the centres practice before participating in TOPSY?
- Was self-management available?
- Have you taught self-management to women before participating in TOPSY?
- IF yes, were there similarities between the way you taught self-management and the TOPSY intervention?

## Health professionals perceptions about woman’s experience of the TOPSY interventions

- Please will you tell me about what went well with the TOPSY interventions. (In each of these areas think about medical management, role management and emotional management)
  - Self-management teaching appointment
  - 2 week phone call
  - Phone helpline
- And what went less well with these things. (In each of these areas think about medical management, role management and emotional management)
  - Self-management teaching appointment
  - 2 week phone call
  - Phone helpline
- Seek an example of a case that went well and what were the features that the HCP attributes to why it was good and an example of a case that didn’t go so well.
- Were there any factors that you think influenced how the intervention was delivered (probe in relation to service structures, factors about the women).

## Following the study protocol

- Please will you tell me what it was like using the study intervention protocols
  - How easy/ difficult were they to follow
    - Did you have to make any variations? What were they?
    - What was it like to complete the paperwork?
    - What was it like to have you session audio-recorded (if it was)
- Please tell me about the training you received prior to taking part in TOPSY?
  - What went well?/ less well?
  - What could have been improved?
- Please will you tell me about the support you received during the study
  - Was this sufficient?
  - What could have been done differently?
- What might you continue doing with women even though the trial is over?

## Expectations of about being in the study

- Please tell me about whether being involved with this study met your expectations?
  - Would you take part in future research studies as a health professional delivering an intervention (with this type of protocol)?
  - Would you recommend involvement in research to other health professionals?
  - Do you think participating in the TOPSY study will change the practice at this centre?

**A9**

**Self-Management Teaching Sessions a priori coding frame**

| **Checklist item** | **Yes/no**  **(Y) / (N)** | **Good quality** | **Limited quality** | **Insufficient quality** | **Not applicable** | **Notes** |
| --- | --- | --- | --- | --- | --- | --- |
| Explained role within TOPSY study |  |  |  |  |  |  |
| Information on organ prolapse provided |  |  |  |  |  |  |
| Talked about taking responsibility for own health |  |  |  |  |  |  |
| Talked about benefits of self-management |  |  |  |  |  |  |
| Talked about managing emotions |  |  |  |  |  |  |
| Discussed anatomy of vagina and pelvis |  |  |  |  |  |  |
| Talked about the type of pessary the woman is using |  |  |  |  |  |  |
| Woman provided with pessary to handle |  |  |  |  |  |  |
| Lubrication demonstration |  |  |  |  |  |  |
| Demonstration of pessary insertion |  |  |  |  |  |  |
| Demonstration of pessary positioning |  |  |  |  |  |  |
| Demonstration of pessary removal |  |  |  |  |  |  |
| Demonstration of pessary cleaning |  |  |  |  |  |  |
| Discussion of pessary storage |  |  |  |  |  |  |
| Discussion on how to receive a replacement pessary |  |  |  |  |  |  |
| Talked about what to do when getting pregnant |  |  |  |  |  |  |
| Talked about what to do in event of problems |  |  |  |  |  |  |
| Support telephone number/video link provided |  |  |  |  |  |  |
| Talked about common problems |  |  |  |  |  |  |
| Woman able to practice pessary removal, insertion and positioning |  |  |  |  |  |  |
| Woman informed about having to remove & reinsert pessary at least once over the next 2 weeks |  |  |  |  |  |  |
| Reminded that woman will receive follow up call in 2 weeks |  |  |  |  |  |  |

**Length of recording:**

Note: Highlighted fields relate to patient’s self-efficacy and role management

**Note: If not demonstrated but only discussed, rate demonstration as limited quality and then add a note about the quality of the discussion.**

**A10**

**Self-management 2 week follow-up telephone call a priori coding frame**

| **Checklist Item** | **Yes (Y)/ No (N)** | **Good quality** | **Limited quality** | **Insufficient quality** | **Not applicable** | **notes** |
| --- | --- | --- | --- | --- | --- | --- |
| Introductions made |  |  |  |  |  |  |
| Reason for call explained |  |  |  |  |  |  |
| Asked if woman was able to remove |  |  |  |  |  |  |
| Asked if woman was able to re-insert pessary |  |  |  |  |  |  |
| Asked if woman was able to position pessary |  |  |  |  |  |  |
| Additional phone call arranged (if not yet been able to change pessary) |  |  |  |  |  |  |
| Additional teaching appointment arranged (if necessary) |  |  |  |  |  |  |
| Asked if woman changed to standard care (where applicable) |  |  |  |  |  |  |
| Asked if the woman is experiencing bothersome vaginal discharge |  |  |  |  |  |  |
| Reminded woman that some discharge is normal due to the use of a pessary |  |  |  |  |  |  |
| Asked if the woman is experiencing bothersome vaginal smell |  |  |  |  |  |  |
| Asked if the woman is experiencing Vaginal Pain |  |  |  |  |  |  |
| Asked if the woman is experiencing other pain |  |  |  |  |  |  |
| Asked if the woman had a Urine infection (requiring antibiotics) |  |  |  |  |  |  |
| Asked if the woman is experiencing Urine incontinence (leakage) |  |  |  |  |  |  |
| Asked if the woman is having difficulty emptying bladder |  |  |  |  |  |  |
| Asked if she’s tried to remove pessary to address this complication as discussed during teaching appointment and self-management leaflet |  |  |  |  |  |  |
| Asked if the woman is experiencing bowel incontinence (leakage) |  |  |  |  |  |  |
| Asked if the woman is having difficulty emptying bowel |  |  |  |  |  |  |
| Asked if she’s tried to remove pessary to address this complication as discussed during teaching appointment and self-management leaflet |  |  |  |  |  |  |
| Asked if the woman is experiencing difficulty having sex |  |  |  |  |  |  |
| Asked if the woman is experiencing pain during sex |  |  |  |  |  |  |
| Asked if the woman’s pessary fell out |  |  |  |  |  |  |
| Asked if gone back to clinic to have another fitted |  |  |  |  |  |  |
| Asked if a pessary is in position now |  |  |  |  |  |  |
| Asked if the woman is experiencing Vaginal non-menstrual bleeding |  |  |  |  |  |  |
| Asked about degree of bleeding |  |  |  |  |  |  |
| Reminded woman that some bleeding is normal when changing the pessary |  |  |  |  |  |  |
| Asked if the woman is experiencing difficulty removing pessary |  |  |  |  |  |  |
| Actions discussed to address complications |  |  |  |  |  |  |
| Actions discussed to encourage woman to re-try and change pessary |  |  |  |  |  |  |
| Reminded about additional available resources |  |  |  |  |  |  |
| Reminded woman to change pessary at least once over the next six months |  |  |  |  |  |  |

Note: Highlighted fields relate to patient’s self-efficacy, emotional and role management

Items highlighted are not on the CRF, but conductive to a positive patient-healthcare provider relationship

Good Quality = The questions is asked exactly as stated on the CRF and if necessary a detailed response is given to the participant.

Limited quality = The question has been modified from the CRF, lumped together or the response from HCP to a participant’s question is not detailed enough.

Insufficient quality = This applies when no judgement can be made about the quality of the exchange because the HCP did not ask a question listed on the CRF.
